# Supplementary material for: Characterization of binding between model protein GA-Z and human serum albumin using asymmetrical flow field-flow fractionation and small angle X-ray scattering
Source: PLoS One. 2020 Nov 24;15(11):e0242605. doi: 10.1371/journal.pone.0242605 (PMC7685474; doi:10.1371/journal.pone.0242605)
Supplement: S1 Fig — Each model has a unique colour and was superimposed automatically using DAMAVER. The filtered average model (damfilt.pdb) from DAMAVER software is shown in red spheres on top of the 19 individual models. (PDF) [file pone.0242605.s001.pdf]

Characterization of binding between model protein GA-Z and human serum albumin using asymmetrical flow field-flow fractionation and small angle X-ray scattering

Jaeyeong Choi[1], Marie Wahlgren[1], Vilhelm Ek[2], Ulla Elofsson[3], Jonas Fransson[2], Lars Nilsson[1], Ann Terry[4] and Christopher A.G. Söderberg[3,*]

[1]*Food Technology, Engineering and Nutrition, Faculty of Engineering LTH, Lund University, PO Box 124, SE-221 00 Lund, Sweden*

[2]*Swedish Orphan Biovitrum AB (Sobi), Stockholm, Sweden*

[3]*RISE Research Institutes of Sweden, Division of Bioscience and Materials, Scheelevägen 27, SE-223 70 Lund, Sweden*

[4]*MAX IV Laboratory, Lund University, P.O. Box 118, Lund 221 00, Sweden*

Corresponding author

E-mail: christopher.soderberg@ri.se

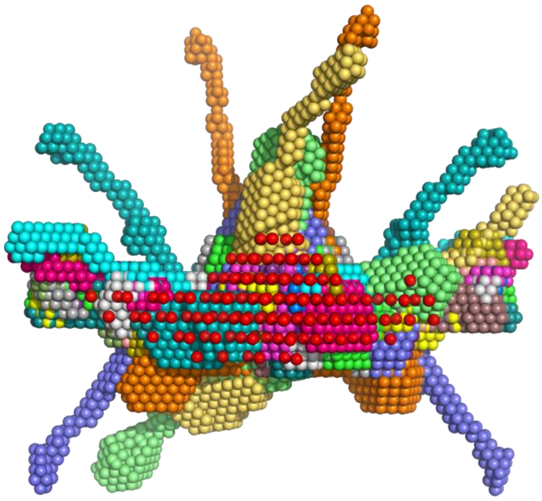

S1 Figure: 19 of the 20 DAMMIF *ab initio* models computed from GA-Z SEC-SAXS data. Each model has a unique colour and was superimposed automatically using DAMAVER. The filtered average model (damfilt.pdb) from DAMAVER software is shown in red spheres on top of the 19 individual models.
