# Supplementary material for: Characterization of binding between model protein GA-Z and human serum albumin using asymmetrical flow field-flow fractionation and small angle X-ray scattering
Source: PLoS One. 2020 Nov 24;15(11):e0242605. doi: 10.1371/journal.pone.0242605 (PMC7685474; doi:10.1371/journal.pone.0242605)
Supplement: Attachment — Submitted filename: Response to Reviewers.docx [file pone.0242605.s002.docx]

We thank the reviewers for their valuable comments to our manuscript.

Please find below our response to reviewers' comments on the manuscript "Characterization of binding between model protein GA-Z and human serum albumin using asymmetrical flow field-flow fractionation and small angle X-ray scattering".

Reviewer #1

1. “What are the binding constants of GA-Z protein with HSA"

It is not possible to determine the binding constant based on the data that we present here. However, we added examples on GA-HSA binding constants in the revised manuscript.

2. “Does protein interaction alter HSA conformation.”

From the SAXS data we cannot see any major alterations to the general shape of HSA, and we have clarified this in the revised manuscript.

3. “What are the binding sites of protein on HSA"

GA-Z binds to the DII domain of HSA, this information has been included in the revised manuscript.

Reviewer #2

1. A more rigorous comparison of the present study with the literature in all respects would enhance the impact of the present study.

The following references have been included to the introduction and discussion parts of the manuscript for comparison. Zorzi *et al.* (2019), Alexander *et al.* (2007), Gapizov *et al.* (2019)

2. The abstract should contain the significance of the results obtained. It is therefore advised to add more on significance at the end of the abstract

The following sentence has been added to the abstract to lift the significance of this manuscript: “Furthermore, GA-Z binds to HSA both as a monomer and a dimer, and this, it can be expected to stay bound also upon dilution following injection in the blood stream.”

3. The manuscript lacks the information on the number of the replicate set of experiments and error analysis.

This has been added to Material and Methods in the revised manuscript.

4. The font size of the text inside the graphs should be increased so that they become more legible. The quality of the figures is not suitable for publication.

We have revised the figures with small fonts to increase their readability. We have also incrased the DPI of low quality figures.

5. In Table. 1 fourth column ‘apparent MW (kDa) from AF4-MALS’. The word apparent should be capitalized.

This has been revised.

6. The presentation and interpretation of results executed should be more vivid.
   In this context, I recommend this article for publishing only after the minor revision.

We have rephrased the discussion of the manuscript in an attempt to make it more “vivid”, although the content have not been altered.

Yours Sincerely

RISE Research Institutes of Sweden AB

__Signature_1	__Signature_2

Christopher Söderberg
